# A novel extraoral ultrasound-guided approach for mandibular nerve block in Rahmani sheep

**DOI:** 10.1186/s12917-024-03924-0

**Published:** 2024-02-24

**Authors:** Mohamed Wefky El-Sherif, Mohamed Ahmed Nazih

**Affiliations:** 1 Department of Surgery, Faculty of Veterinary Medicine, New Valley University, El Kharga, 72511 New Valley Egypt; 2 Department of Anatomy, Faculty of Veterinary Medicine, New Valley University, El Kharga, 72511 New Valley Egypt

**Keywords:** Extraoral, Sheep, Mandibular nerve, Nerve block, Ultrasound guided

## Abstract

**Background:**

Regional anesthesia is the favored choice in ruminant animals compared to general anesthesia, primarily due to its high safety margin and reduced demand for cost-intensive equipment in addition to its field applicability. Ultrasound-guided nerve block has gained significant attention in the field of veterinary medicine. This study utilized twenty-seven sheep of the native Rahmani breed to both demonstrate and evaluate the effectiveness of the ultrasound guided inferior alveolar nerve block technique.

**Methods:**

The research comprised three phases: Phase 1 involved an anatomical examination of mandibles and sheep heads to locate the mandibular foramen and delineate the mandibular nerve course. Phase 2 included ultrasound-guided injection of methylene blue dye at specific sites along the mandibular nerve in cadaveric sheep heads. In Phase 3, clinical implementation of ultrasound-guided inferior alveolar nerve blocks was conducted in 27 live sheep, assessing efficacy, onset, and duration.

**Results:**

Vertical extraoral ultrasound-guided nerve block was achieved successfully in 25 sheep (98%). A preliminary cadaveric study showed good distribution of the injectate at the mandibular nerve site. The mean onset time was 138 ± 18 s, and the mean duration time was 54 ± 4.1 min. Prominent analgesia of the ipsilateral mandible, medial surface of the cheek, and lateral border of the tongue was observed.

**Conclusions:**

Ultrasound-guided mandibular nerve block holds promise as a technique for providing effective and safe anesthesia in sheep undergoing mandibular procedures.

## Background

The mandibular nerve, also known as the inferior alveolar nerve, a branch of the trigeminal nerve, provides sensory innervation to the lower jaw [1]. Blocking this nerve can provide effective analgesia for procedures involving the mandible, such as dental extraction, mandibulectomy, periodontal surgery, endodontic procedures, and jaw fracture repair [1–3]. Several techniques for mandibular nerve block are recognized in animals. Extraoral blocks have been reported in canines [4], felines [4], cattle and equines [5, 6]. Intraoral blocks have been described in canines [4], felines [7], cattle [8, 9] and equines [6]. Traditional methods of performing a mandibular nerve block rely on anatomical landmarks and can be challenging due to individual anatomical variations [10, 11]. The use of ultrasound guidance can help overcome these challenges by allowing real-time visualization of the nerve and surrounding structures, thereby increasing the accuracy of the block and potentially reducing the risk of complications [12–16].

In human medicine, the technique involves identifying the pterygomandibular space between the coronoid process and the condyle of the mandible using ultrasound [17]. The needle is then guided to the target area around the maxillary artery, and the anesthetic agent is administered [18–20]. Ultrasound-guided mandibular nerve block was reported in donkeys [21], cadavers of horses [22] and exotic animals [23].

Transforming this technique to veterinary medicine, particularly in sheep, could offer several benefits. For instance, it could improve the welfare of the animals by providing more effective pain management. It could also potentially reduce the risk of complications associated with blind nerve blocks, such as nerve damage or intravascular injection. The aim of this study was to describe a novel extraoral ultrasound-guided mandibular nerve block in sheep.

## Methods

The research investigation comprised three distinct phases. In Phase 1, an anatomical examination of twenty-five mandibles and the surgical anatomy of the mandibular region in five sheep heads was undertaken to locate the mandibular foramen and delineate the course of the mandibular nerve. Phase 2 involved the ultrasound-guided injection of a 1:1 mixture of 2 cc of methylene blue dye and sterile isotonic saline solution at the ultrasound visualized nerve site. Ten injections were performed along the mandibular nerve of each side in five sheep heads. Phase 3 encompassed the clinical implementation of ultrasound-guided inferior alveolar nerve blocks in a cohort of 27 live sheep, consisting of 24 females and 3 males.

### Phase 1: anatomical study

**Fig. 1:**
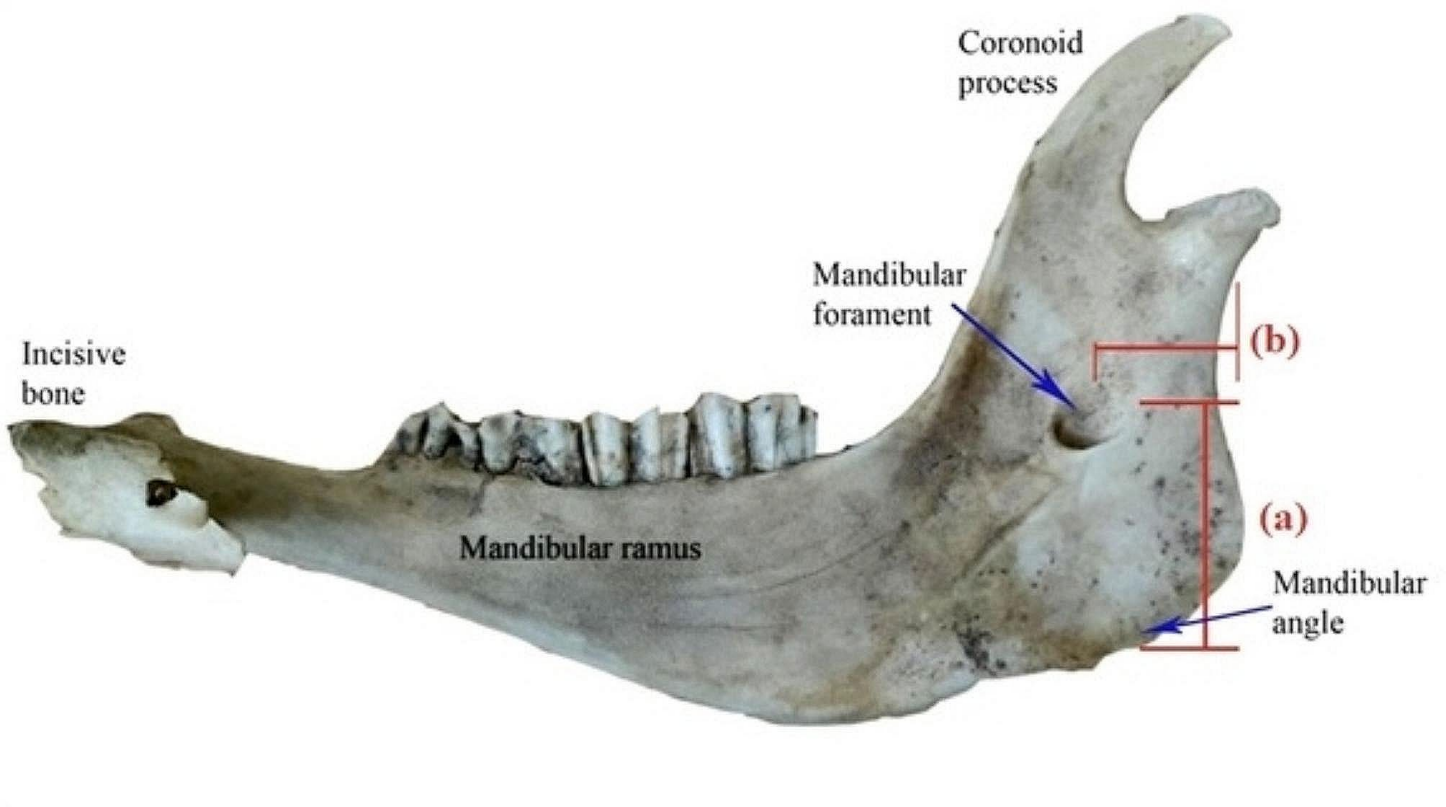
A photograph showing the right half of the mandible. (Medial view). (**a**) the distance from the mandibular angle to the mandibular foramen. (**b**) the distance from the caudal mandibular border to the mandibular foramen

In this study phase, 25 mandibles of both sexes were sourced from two distinct locations: the faculty of veterinary medicine morgue at New Valley University. These specimens were utilized for acquiring measurements, specifically to determine (a) the mean distance between the mandibular angle and the mandibular foramen and (b) the mean distance between the posterior border of the ramus and mandibular foramen, as illustrated in Fig. 1. Measurement procedures were carried out employing a Vernier caliper. The results of this investigation were used as reference values for the second phase of the study.

### Phase 2: ultrasound-guided inferior alveolar nerve block: cadaveric study

**Fig. 2:**
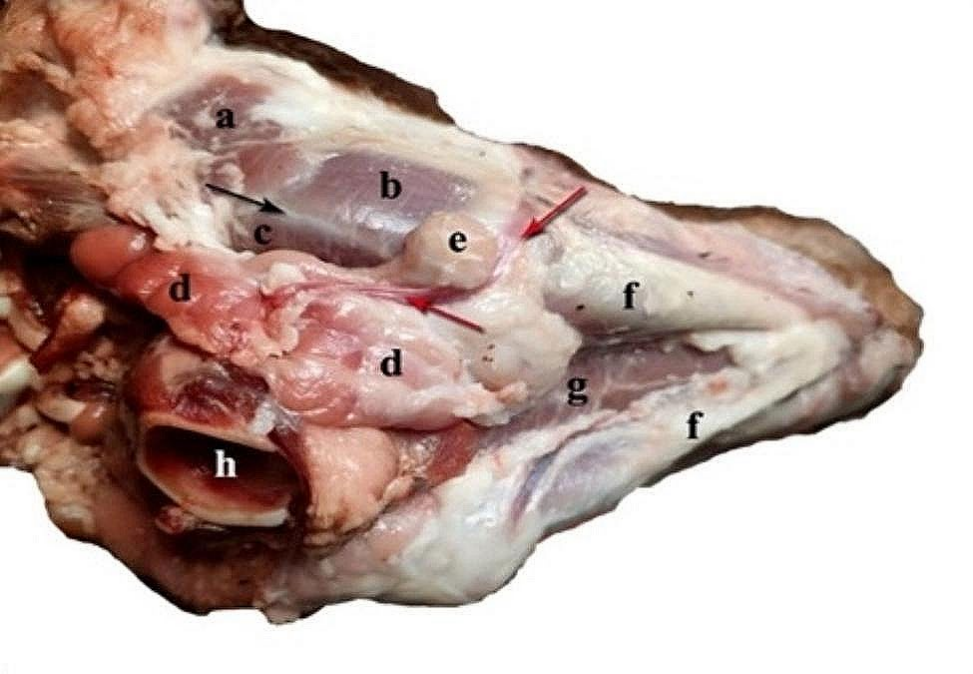
A photograph showing the superficially dissected head of sheep (ventral view). a-Parotid salivary gland, b- Masseter muscle, c- Pterygoideus medialis muscle, d- The mandibular salivary gland, e- The mandibular lymph node, f- Mandibular ramus, g- Mylohyoideus muscle, and h- Larynx. The black arrow indicates the mandibular angle. The red arrows indicate the lingo-facial vein

Six freshly acquired cadaveric heads from adult Barki sheep were sourced from a local slaughterhouse for this study. One of these heads was dedicated to an in-depth dissection of the intermandibular space, offering a comprehensive description of the anatomical structures traversed by the needle during the needed block. The remaining five heads served a dual purpose: first, to gather information about the superficial anatomy of the mandibular region and intermandibular space, as depicted in Fig. 2, and second, to delineate the optimal positioning of the ultrasound probe and acoustic window. Following these observations, the heads were then utilized for further investigations in the ultrasound-guided mandibular nerve block cadaveric study.

**Fig. 3:**
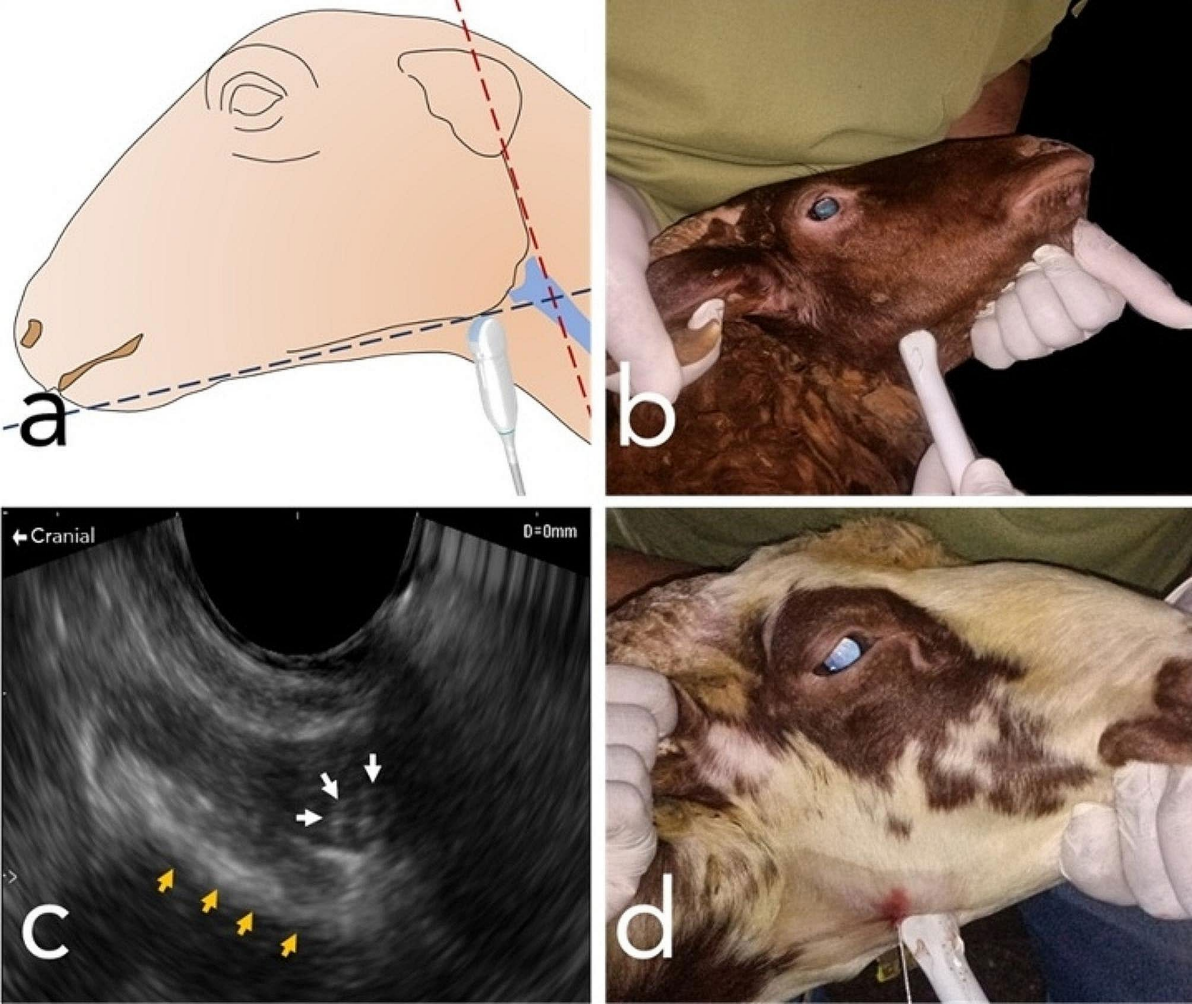
Slide showing the procedure of ultrasound guided inferior alveolar block. (**a**) transducer placement, (**b**) short axis ultrasonography of the inferior alveolar nerve, (**c**) the inferior alveolar nerve appeared as spherical echogenic structure with honeycomb shape, (**d**) needle insertion under guidance of ultrasound and injection process

The localization of the mandibular nerve was accomplished using a micro convex ultrasound probe (Edan DUS 60, Edan, China). A reference system was established with the delineation of an imaginary red line representing the caudal border of the mandible and another imaginary blue line signifying the ventral border of the mandible (Fig. 3). The ultrasound settings were configured to emulate those typically employed for vascular examination, with a maximum depth of 60 mm and a frequency of 9.4 MHz. The probe was positioned perpendicularly and with a slight lateral tilt at the intermandibular space, specifically situated 1.5 cm cranially from the red line demarcating the caudal border of the mandible (Fig. 3). The inferior alveolar nerve presented as a cross-sectional honeycomb appearance and was anatomically situated medially within the ramus mandible (Fig. 3). A 22G, 90 mm spinal needle was percutaneously introduced and meticulously advanced to the predetermined nerve site, guided continuously by ultrasound imaging. Subsequently, a solution comprising 2 cc of methylene blue dye was introduced via injection. This procedural sequence was replicated bilaterally and across the remaining four sheep heads, yielding a total of ten distinct procedures. Following the administration of injections, the medial mandibular region underwent meticulous dissection to assess the precision of the injections, as indicated by the distribution of the dye within the nerve. The radius of dye dispersion was systematically documented to facilitate subsequent analytical evaluation.

### Phase 3: ultrasound guided inferior alveolar nerve block: clinical study

Twenty-seven sheep (24 females and 3 males) were utilized in this study. Unilateral ultrasound-guided mandibular nerve block procedures were carried out on all participating animals (Fig. 3-b). The animals were physically restrained in a standing position with the head extended on a side, and the intermandibular space was meticulously cleansed and disinfected by 70% alcohol swans and chlorhexidine solution (Cyteal, MUP, Egypt).

### Creation of the ultrasound window and probe position

The ultrasound transducer was positioned 1.5 cm cranially to the posterior border of the ramus mandible, perpendicular to the intermandibular floor of the mouth. Slight tilting of the transducer medio-lateral was employed to optimize the visualization of the mandibular nerve. A 22G, 90 mm spinal needle was percutaneously inserted and carefully advanced under ultrasound guidance to reach the nerve location. Subsequently, 2 cc of 2% lidocaine HCl solution (Lidocaine 2%, Hospira Inc., USA) was injected, and then the needle was withdrawn slowly.

### Assessment and Data Collection

The mean time to locate the nerve was assessed. The starting point was determined as the duration from initiating the ultrasound probe application to the designated examination area, while the endpoint was identified as the moment when the mandibular nerve was successfully located. The evaluation of nerve block onset by pinprick testing [24], duration, and efficacy involved the assessment of behavioral indicators and pain scores. An ipsilateral mandibular superficial and deep pinprick examination was performed to gauge the extent of analgesia. The pinprick testing was applied to the gum at different levels of medial and lateral borders of the mandibular body starting from caudal to rostral pattern. The extent of the block was evaluated through the response of the sheep to superficial or deep pinpricks and caudal to rostral desensitization. The success of the block was evaluated by the complete lack of pain response recognized by absence of vocalization, and resistance during the testing. Observations were performed immediately after the injection and at regular intervals (every 10 min), and pain scores were recorded using a modified visual analog scale [25]. The scale ranged from 0 (indicating no pain, characterized by no reactive response to the pinprick), 1 (indicating diminishing pain, recognized by a slight response to deep pinprick), 2 (indicating pain manifestation in response to deep pinprick), to 3 (indicating severe pain, recognized as a strong reactive response to superficial pinprick). The evaluation process was performed blindly by an independent surgeon. The onset of the block was defined as the time of complete loss of sensation of the gums against deep pinprick testing. The recovery point was identified when the sheep began to respond to the pinprick test with head withdrawal and vocalization, signifying the resolution of the nerve block effect.

### Statistical analysis

Data were analyzed using appropriate statistical methods, including t tests and analysis of variance (ANOVA), to compare the effectiveness of the ultrasound-guided mandibular nerve block with the blind nerve block technique. Statistical significance was set at *p* < 0.05. The statistical analysis was conducted using the SPSS software (version 20.0; IBM, America).

## Results

### Dissection findings

The mean distance between the mandibular foramen and the mandibular angle was approximately 4.5 ± 0.3 cm, and the mean distance between the posterior border of the mandible ramus and the mandibular foramen was 1.9 ± 0.2 cm.

**Fig. 4:**
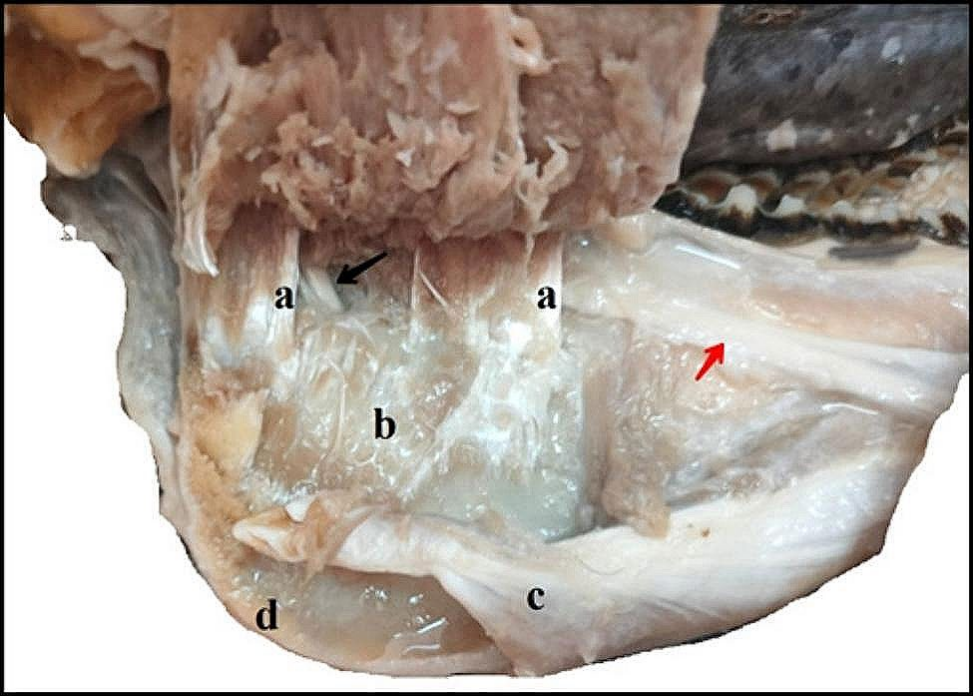
A photograph showing the intermandibular space (left ramus). a- The muscular attachment of the pterygoideus medialis muscle, b- Pterygoideus medialis fossa, c- Rostral belly of digastricus muscle, d- Mandibular angle. The black arrow indicates the mandibular alveolar nerve. The red arrow indicates the lingual branch of mandibular nerve. The bluish colored area indicates the infiltrated injected dye

The surgical approach encompassed the anatomical dissection of the lateral laryngeal region, extending from the mandibular angle laterally to the larynx medially. Superficially, this space is occupied by the mandibular salivary gland, bridging the gap between the larynx and the mandibular angle. Positioned on the ventro-lateral and rostral aspect of the gland, the mandibular lymph node takes on an ellipsoid or spherical morphology. The lingo-facial vein divides between the gland and the lymph node, running along the rostral aspect of the masseter muscle, as depicted in Fig. 4. A deeper dissection of the region reveals its lateral border, defined by the rostral belly of the digastricus muscle, and its medial border, delineated by the laryngeal muscles.

**Fig. 5:**
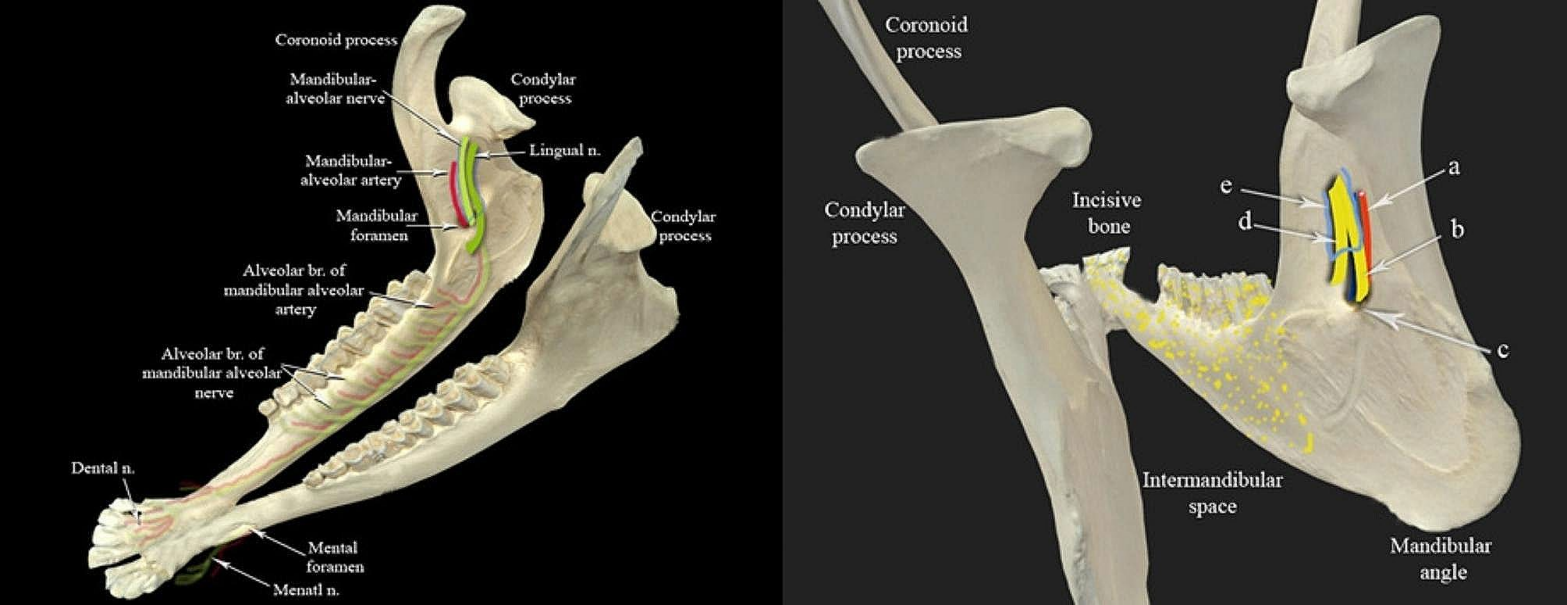
A photograph showing the mandible (dorso-lateral view) demonstrates the passage and termination of the mandibular alveolar nerve. (**a**) Mandibular alveolar artery, (**b**) Mandibular alveolar nerve, (**c**) Mandibular foramen, (**d**) The lingual nerve, and (**e**) Common sheath for mandibular alveolar and lingual nerves

The mandibular nerve, as illustrated in Fig. 5, shares a common sheath with the lingual branch of the trigeminal mandibular nerve. This sheath descends in a rostro-ventral direction along the lateral aspect of the pterygoideus medialis muscle. The lingual nerve subsequently detaches rostrally from the sheath, dorsal to the mandibular foramen, at an approximate distance of 0.7 ± 0.1 cm. Descending ventrally, the nerve courses across the ventral border of the styloglossus muscle, from where it gives rise to multiple branches supplying the anterior two-thirds of the tongue. The mandibular alveolar nerve perforates the mandibular foramen, passes through the mandibular canal, and ultimately emerges rostrally as the mental nerve, which distributes within the submental region and lower lip tissue.

### Ultrasound guided mandibular nerve block: cadaveric study

**Fig. 6:**
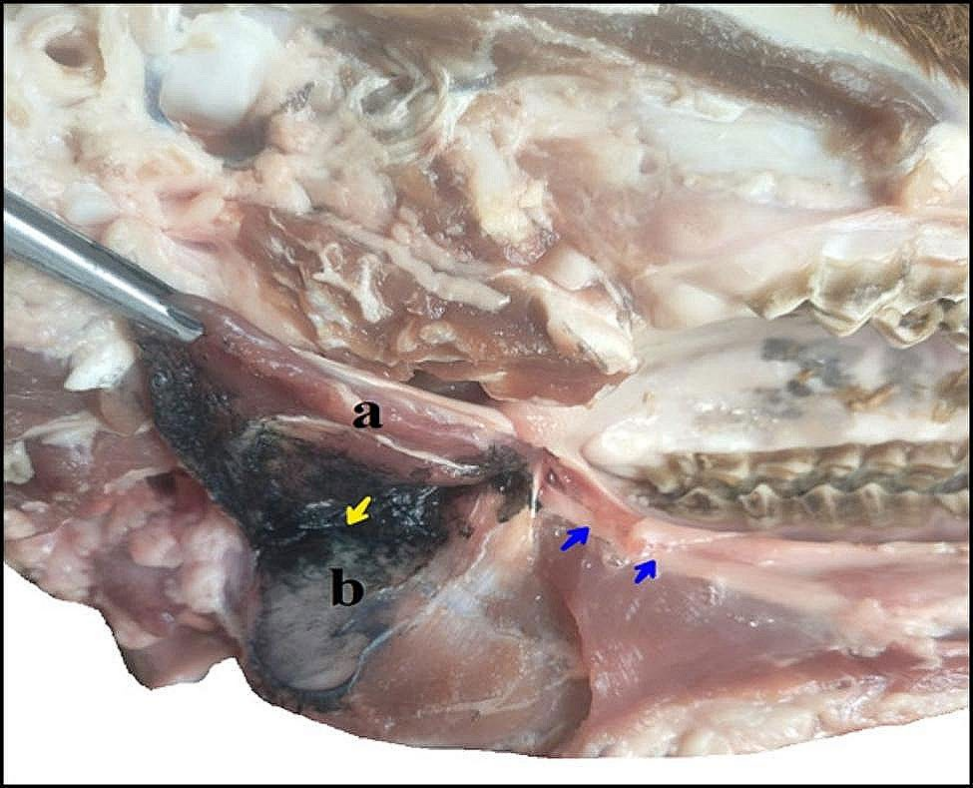
Dissection of specimens after methylene blue injection. (**a**) pterygoideus medialis muscle, (**b**) pterygoideus fossa, yellow arrow indicates the mandibular nerve, the blue arrows indicate the lingual branch

Determination of the mandibular nerve was successful in all 5 cases. The mean time elapsed to locate the nerve was 3 ± 0.05 min. The five mandibular nerves were dyed methylene blue with variable degrees of radial diffusion. The mean value of the dye diffusion radius was 48 ± 27 mm (Fig. 6).

### Ultrasound guided mandibular nerve block: clinical study

**Fig. 7:**
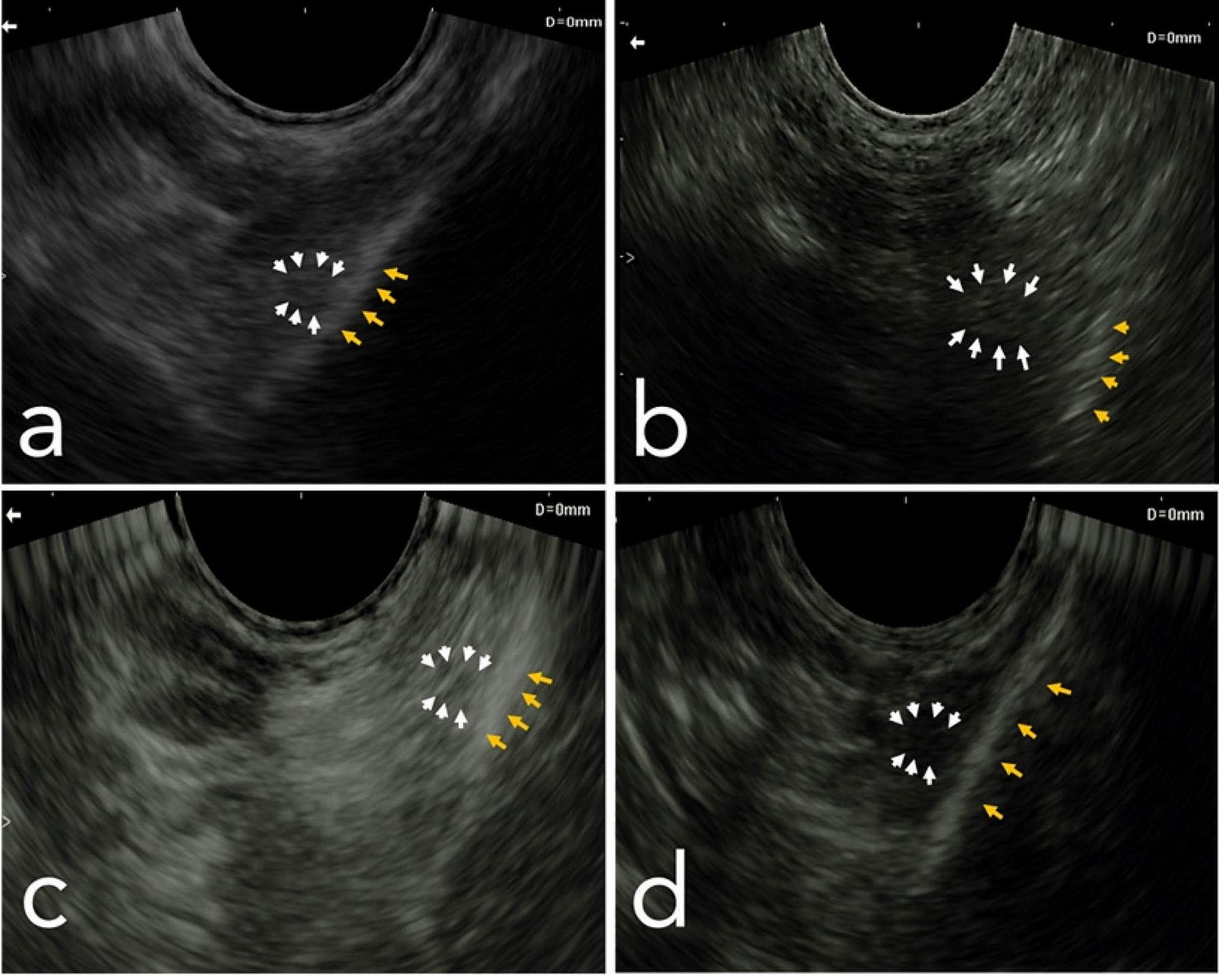
A slide showing different views of the inferior alveolar nerve and its relation to the ramus mandible. The white arrows indicate the nerve, and the yellow arrows indicate the mandible. (**a**) The figure illustrates the characteristic ultrasonographic honeycomb shape of the mandibular nerve. (**b**, **c** and **d**) The figures depict the mandibular nerve, visualized as a spherical echogenic structure

Locating the nerve time varied among animals, and the mean time was 6 ± 1.32 min. The nerve was determined according to its sonographic anatomy and location on the medial surface of the ramus mandible. In more relevant images, the nerve appeared as a honeycomb sphere (Fig. 7-a); in other images, the nerve appeared as an echogenic sphere (Fig. 7-b, c, and d).

The success rate of locating the nerve ultrasonography in 25 sheep (98%) was within an acceptable procedural time (15 min), and the other 2 (2%) sheep were discarded due to discomfort and painful restraint. Ipsilateral mandibular analgesia was successfully applied in all participating sheep with score (0) analgesia. The analgesia encompasses the mandible and partially the ipsilateral cheek and lateral side of the tongue. The mean onset time was 138 ± 18 s. The mean duration time was 54 ± 4.1 min.

## Discussion

The use of ultrasound-guided techniques in veterinary medicine has been on the rise due to the increased precision and safety they offer [12, 13, 26, 27]. One such application is ultrasound-guided mandibular nerve block. This technique is particularly useful in procedures involving the mandible, where effective anesthesia is crucial for successful surgery and pain management.

The authors succeeded in achieving accurate perineural injection of the mandibular nerve using an ultrasound-guided vertical extraoral approach in sheep. The results of the current investigation showed that the ultrasound visualization window of the mandibular foramen with a micro convex transducer was achievable through the intermandibular space, the endo-cavity probe provided the operator good handling, and needle adjustments were feasible.

Regional anesthetic techniques require an understanding of the anatomical structure, and the challenges that may operators face are identifying precise anatomical landmarks. Heterogeneity in individual anatomy can result in suboptimal success rates for specific peripheral nerve blocks [28]. The results of our study showed that ultrasound guidance with proper probe positioning facilitated accurate deposition of the anesthetic solution at the nerve site and subsequent competent analgesia. In this investigation, sedatives were not used to avoid masking effects and to accurately measure the anesthetic values.

The potential for inadvertent penetration into adjacent structures is reported in blind techniques [29]. The ultrasound-guided approach described here offers real-time visualization of anatomical landmarks for precise localization and hence a low incidence of faulty injection.

Administration of large doses of local anesthetic solution may encounter mechanical, ischemic, or inflammatory symptoms [30, 31]. The local anesthetic volume of 2 ml used in this study was adequate to produce satisfactory analgesia in all participating animals. Earlier studies demonstrated the administration of 1.4 ml [29] up to 2 ml [7] of 0.5% anesthetic solution in dogs.

Regarding the cadaveric study performed in this study, the difference may be attributed to variations in diffusion distances between dye solution and local anesthetic solution due to their distinct physical characteristics. Although this method is extensively used to assess accuracy [8, 32–34], computed tomography assessment is generally regarded as the gold standard and is likely representative of a clinical scenario, as stated by other authors [3, 35].

Notably, there was notable lingual nerve desensitization, a result that was consistent with the cadaveric study performed here and findings reported by JP Johnson, RK Peckham, C Rowan, A Wolfe and JM O’Leary [34]. Consequently, bilateral mandibular nerve block using this approach should be avoided to minimize the risk of lingual laceration following bilateral desensitization, as reported by G Kwon and MH Hohman [36].

Complications of mandibular nerve block may encompass infection of the anesthesia site, abscess formation, hematoma, vessel puncture, nerve damage, and neuritis [36]. In our study, a short follow-up period did not reveal any complications. This may be due to the small volume of anesthetic used, and ultrasound guidance diminishes the injection needle injury to the nerve and subsequent complications.

Despite the potential benefits, the use of ultrasound-guided mandibular nerve block in sheep has not been reported in the literature. This could be due to a lack of familiarity with the technique among veterinarians or limited access to ultrasound equipment in some settings. However, with the increasing availability of portable ultrasound machines and the growing interest in ultrasound-guided techniques, it is likely that this technique will become more prevalent in the future.

However, further research is needed to establish the efficacy and safety of ultrasound-guided mandibular nerve blocks in sheep. Factors to consider include the choice of ultrasound equipment, the optimal approach for visualizing the mandibular nerve in sheep, and the most effective anesthetic agents, and more research is needed to fully understand their potential benefits and limitations. Given that the experiment was conducted on ostensibly healthy animals, conducting additional research would be of substantial importance to investigate the ultrasound-guided mandibular block in sheep afflicted with clinical mandibular surgical conditions.

## Conclusions

In conclusion, the ultrasound-guided vertical extraoral approach offers improved visualization of anatomical landmarks for precise injection localization to provide effective and safe anesthesia in sheep undergoing mandibular procedures.

## Data Availability

Data is available from the corresponding author upon request.
